# Behavioral Sleep Treatment in ADHD Across Child and Adolescent Development: Recent Findings, Integration with Existing Interventions, and Emerging Directions

**DOI:** 10.1007/s40675-026-00387-7

**Published:** 2026-06-20

**Authors:** Stephen P. Becker, Alison E. Pritchard, Jessica R. Lunsford-Avery

**Affiliations:** 1 Division of Behavioral Medicine and Clinical Psychology, Cincinnati Children’s Hospital Medical Center and Department of Pediatrics, University of Cincinnati College of Medicine, 3333 Burnet Ave., MLC 10006, Cincinnati, Ohio 45229, USA; 2 Center for Neuropsychological and Psychological Assessment, Kennedy Krieger Institute and Department of Psychiatry and Behavioral Sciences, Johns Hopkins University School of Medicine, Baltimore, USA; 3 Department of Psychiatry and Behavioral Sciences, Duke University School of Medicine, Durham, USA

**Keywords:** Attention-deficit/hyperactivity disorder, Circadian, Intervention, Sleep, Treatment

## Abstract

**Purpose of Review:**

Sleep disturbances are highly prevalent among youth with attention-deficit/hyperactivity disorder (ADHD) and contribute to emotional, cognitive, and functional impairments. Although historically under-addressed in ADHD treatment, there is increasing recognition of sleep as a critical intervention target. This paper synthesizes recent advances in behavioral sleep interventions for youth with ADHD, emphasizing developmental considerations spanning early childhood, middle childhood, and adolescence.

**Recent Findings:**

Across developmental periods, growing evidence supports the feasibility and effectiveness of behavioral sleep interventions, particularly for improving parent- and self-reported sleep outcomes. We also examine the limited literature on integrating sleep-focused strategies into established evidence-based behavioral treatments for ADHD, highlighting opportunities and challenges for embedding sleep within existing care models.

**Summary:**

We identify key emerging directions, including the need for developmentally-tailored and mechanistically-informed interventions, increased focus on circadian and sleep regularity processes, and scalable delivery approaches leveraging digital and integrated care settings. Advancing this work holds promise for improving both nighttime sleep and daytime functioning for youth with ADHD and their families.

## Introduction

Seventy years ago, it was already apparent that children with hyperkinetic behavior syndrome, a precursor to the current diagnosis of attention-deficit/hyperactivity disorder (ADHD), experience pervasive and impairing sleep disturbances. Based on their clinical experiences of hyperactive and distractible children at the Emma Pendleton Bradley Home, Laufer and Denhoff [1] observed that “generally, the parents are so desperate over the night problems that the daytime ones pale in comparison” (p. 465). And yet, interventions developed and tested for these children over subsequent decades focused almost exclusively on their daytime difficulties, ignoring sleep problems entirely or relegating them to an afterthought or potential medication side effect to be monitored [2]. Appreciably, the tide has started to turn, with increasing calls to recognize ADHD as a 24-hour disorder [3] that necessitates assessing and treating sleep problems in youth with ADHD [2, 4].

Although rates vary widely depending on participant characteristics and sleep measurement methods [5-7], up to 70% of youth with ADHD experience sleep problems [8, 9], with potentially higher prevalence in girls than boys with ADHD [9, 10]. A recent study of over 25,000 U.S. children (ages 6–11 years) found 45% of children with an ADHD diagnosis had short parent-reported sleep duration compared to 34% of children without ADHD, with moderate/severe ADHD associated with higher odds of having short sleep duration compared to those with mild symptoms [11]. In adolescence, the odds of being classified with clinically elevated sleep disturbance is 7.31 times greater in adolescents with ADHD compared to adolescents without ADHD [12]. Moreover, although the risk of being diagnosed with a sleep disorder is elevated across the lifespan for individuals with ADHD compared to those without, and the overall *prevalence* of having any sleep disorder is highest in adults with ADHD, the *relative risk* of having a sleep disorder diagnosis is highest among children and adolescents with ADHD compared to those without ADHD [10]. This suggests that, compared to their peers, youth with ADHD are especially likely to have an identified sleep disorder, likely due to both genetic and environmental influences [13]. When present, insufficient or poor sleep is associated with increased daytime impairment in youth with ADHD, including greater irritability and internalizing symptoms [14-16], poorer neurocognitive performance [17], and lower quality of life [8, 18].

High rates of sleep problems, coupled with their impact on daytime functioning, underscore the importance of treating sleep in youth with ADHD. In this narrative review we (1) briefly review recent findings of sleep-focused behavioral interventions for youth with ADHD, (2) advance possibilities for incorporating sleep intervention strategies into existing behavioral interventions for youth with ADHD, and (3) highlight emerging directions in this area. Throughout, a developmental approach is emphasized given the importance of considering differences in both ADHD and sleep that unfold across early childhood, middle childhood, and adolescence.

### Recent Findings of Sleep-Focused Interventions for Youth with ADHD

Recent systematic and meta-analytic reviews provide comprehensive information regarding effects of behavioral sleep interventions for children and adolescents with ADHD [19, 20]. Accordingly, in this section we highlight key findings, including studies ineligible for inclusion in those systematic/meta-analytic reviews or published more recently and studies focused on other neurodevelopmental conditions when data specific to ADHD are lacking.

#### Early Childhood

Although most children with ADHD are not diagnosed until the school-aged years, difficulties with regulating attention, hyperactivity, and impulsivity are often apparent in early development [21, 22]. Furthermore, the onset of ADHD symptoms during the preschool years contributes to delayed development of age-expected skills (i.e., pre-academic learning, increased autonomy in daily routines, establishment of peer relationships), which in turn increases risk of worsened ADHD prognosis, executive functioning challenges, and psychosocial impairment across the lifespan [23]. Sleep disturbances are prevalent among preschoolers with elevated ADHD symptoms and may play a key role in increasing ADHD-related impairments [24]. A recent review found cross-sectional evidence for associations between ADHD symptoms and shorter sleep duration, longer sleep onset latency, greater nocturnal awakenings, and daytime sleepiness in preschoolers [25]. Moreover, longitudinal research suggests that sleep problems are bidirectionally related to ADHD symptoms across early development [26, 27], and further, that sleep disturbances in the toddlerhood/preschool years predict ADHD symptoms across adolescence and into adulthood [28, 29].

Although there is no current literature to inform the utility of behavioral sleep interventions among preschoolers with ADHD, there is strong rationale for investigating treatment of sleep disturbances in early development as a method to improve ADHD trajectories [24]. First, early development is characterized by high neural plasticity and sleep actively supports development of executive functions and emotional regulation during this period [30, 31]. Among preschoolers with elevated risk for ADHD, treating sleep disturbances early in life may therefore provide a critical opportunity to effect long-lasting improvements in functional outcomes. Second, although not in an ADHD sample, a 5-session behavioral sleep intervention for preschoolers with sleep complaints has been shown to improve sleep, behavioral problems, and externalizing symptoms, with sustained effects up to one year later [32]. Moreover, improvements in sleep following this intervention, which included standard pediatric sleep strategies (e.g., psychoeducation, sleep hygiene, bedtime routines) as well as specific strategies for bedtime fears (i.e., exposure therapy) and bedtime resistance (e.g., effective instructions, time-out), were found to mediate improvements in externalizing symptoms at subsequent follow-ups.

Extending to preschoolers with neurodevelopmental conditions, similar findings were observed in a test of a 3-session behavioral sleep intervention among preschoolers with autism spectrum disorder, which also found improvements in sleep problems, sleep onset delay, bedtime resistance, and behavioral and emotional functioning compared to treatment as usual, most notably in regard to reductions in externalizing symptoms [33]. Similar to above, this intervention also focused on standard pediatric sleep strategies, but with some modifications for the ASD population, and included psychoeducation about sleep and sleep disturbances in ASD, sleep hygiene strategies, bedtime routine paired with visual cues, bedtime fading, stimulus control, graduated extinction, rewards for bedtime behaviors, and relaxation techniques. Taken together, studies highlight the potential utility of behavioral sleep improvements for improving clinical trajectories among preschoolers with ADHD.

#### Middle Childhood

Over the past 5 years, several systematic reviews and meta-analyses have summarized the existing evidence for behavioral sleep interventions in children between the ages of 5 and 12 (i.e., middle childhood). The most recent review, published in 2026, includes 15 randomized controlled trials (RCTs) of behavioral sleep interventions, which are broadly defined to include traditional therapeutic sleep interventions, as well as alternative interventions such as weighted blankets and cognitive training [34]. Also included are behavioral interventions for ADHD that do not have a sleep-specific component. This meta-analysis found significant effects of behavioral sleep interventions on self-reported sleep duration and parent-reported sleep disturbance, but not on objectively-measured total sleep time, sleep efficiency, or sleep onset latency [34]. Published in 2024, another review of RCTs of interventions for sleep disorders among children with ADHD found 10 studies of nonpharmacological interventions [2]. Behavioral sleep interventions were found to be more effective than treatment as usual in improving parent-reported sleep problems, as were psychoeducation and sleep hygiene training. Reviews from 2022 [20] and 2023 [19] offer similar take-home messages: behavioral treatments for sleep seem to improve parent-reported sleep problems for children with ADHD; however, their impact on objective sleep measures remains unclear (and understudied).

Two behavioral sleep interventions have been specifically designed for children with ADHD. Sleeping Sound [35], developed in Australia, is a brief intervention that can be delivered effectively by pediatricians as well as psychologists [36], in-person or remotely [37]. The intervention is comprised of two sessions conducted with the child and their parent(s). The first 30–60 min session involves assessment of sleep concerns, psychoeducation around sleep and sleep hygiene, and developing an individualized plan targeting the child’s specific sleep problems. The plan is developed using standardized strategies based on the nature of the problem – for instance, strategies designed to address bedtime resistance include bedtime fading, establishing an enjoyable bedtime routine, planned ignoring, and rewarding desired behaviors. Two weeks later the second session reviews and reinforces strategies, progress monitoring, and troubleshooting. A brief follow-up call is optional two weeks later for further progress monitoring and strategy reinforcement. Across multiple RCTs, Sleeping Sound has demonstrated both efficacy and effectiveness, showing a positive impact on parent-reported sleep, quality of life, and ADHD symptoms, as well as on some actigraphy metrics [35-39].

Better Nights, Better Days [40] is another behavioral sleep intervention for children with ADHD that was developed with the goal of being maximally accessible to patients and families. It is delivered entirely remotely, with only the support of a paraprofessional coach via telephone. Families participating in Better Nights, Better Days receive a written intervention manual detailing 5 sessions that include psychoeducation around sleep, helping parents maximize their child’s environment to support sleep, and helping them create routines and schedules for sleep with their child. Focusing on difficulties with sleep onset, the intervention also instructs parents in using bedtime fading with response cost and positive reinforcement. The paraprofessional coach guides parents through each session by phone. A RCT of Better Nights, Better Days found that the intervention led to improvements in parent-rated sleep problems and psychosocial functioning that were sustained 6 months after treatment [40]; furthermore, latency to sleep onset improved by objective measurement [40].

Five unnamed behavioral sleep interventions have also been tested recently with children with ADHD – one in Egypt [41], one in India [42], and three in Iran [43-45]. All were developed based on the existing behavioral sleep intervention literature. The structure of treatment varied from one intervention to the next, in terms of in person vs. remote administration, session length and frequency, and type of clinician delivering the intervention. Intervention components also varied across treatments, with all including psychoeducation around sleep and sleep hygiene, and most including training in the principles of behavioral modification. Some of these interventions also included discussion of controlling environmental stimuli, bedtime fading, relaxation techniques, and/or establishing consistent schedules. RCTs testing all five interventions found that they all resulted in significant improvements in parent-rated sleep problems, with three additionally improving child behavior, and two positively impacting quality of life [41-43].

Finally, one intervention for children with ADHD focused on sleep hygiene and progressive muscle relaxation [46]. Conducted in Turkey, this study compared sleep hygiene training for parents alone to this training plus progressive muscle relaxation training for children. In an RCT, both intervention groups showed significant gains in parent-reported child and parent sleep, as well as ADHD symptoms and performance-based measures of attention; however, the combined treatment demonstrated improvement over sleep hygiene alone in terms of selective attention, peer difficulties, and anxiety [46].

#### Adolescence

During adolescence, the interplay of psychosocial and societal pressures alongside sleep regulatory maturation has been termed a “perfect storm” contributing to short and misaligned sleep [47]. There is a small but growing body of evidence supporting cognitive-behavioral treatment (CBT) sleep interventions in adolescents with sleep and circadian problems [48, 49]. A three-week experimental sleep restriction/extension study found that during extended sleep, compared to restricted sleep, adolescents with ADHD experienced improved ADHD severity, lower depressive symptoms and oppositional behaviors, and improved affective functioning [14, 15], providing causal evidence that targeting sleep improves sleep as well as daytime functioning in adolescents with ADHD. Further, two recent interventions have been tested to evaluate the feasibility and effects of cognitive-behavioral sleep treatments in adolescents with ADHD and co-occurring sleep problems [50, 51].

As its name implies, the Transdiagnostic Intervention for Sleep and Circadian Dysfunction (TSC) was designed to treat a range of sleep disturbances in a range of clinical populations [49]. As an initial test of this in adolescents with ADHD, a pilot open trial examined TSC in a sample of 14 adolescents (ages 13–17 years) with ADHD and sleep problems (defined as short sleep duration, poor overall sleep quality, and/or strong eveningness chronotype) [50]. TSC was feasible to implement, viewed as highly acceptable to adolescents and their caregivers, and demonstrated strong preliminary support for effectiveness. Specifically, adolescents had improvements in sleep (particularly questionnaires, with mixed findings with daily diary and actigraphy measures), and the percentage of participants classified as poor sleepers dropped from 71% pre-intervention to 21% and 29% following intervention and at three-month follow-up, respectively [50]. Improvements were also seen in mental health symptoms (ADHD and co-occurring depressive and cognitive disengagement syndrome symptoms) and daily life executive functioning from pre- to post-intervention, with many improvements maintained at follow-up, though effects were modest for anxiety and no improvements were observed for oppositional defiant symptoms or emotion dysregulation [50]. Importantly, this study was not a randomized design and so precludes causal inferences, but the promising findings point to the need for larger randomized trials to rigorously test the efficacy and effectiveness of TSC in adolescents with ADHD.

Unlike Becker et al., who took the existing TSC intervention and tested it in adolescents with ADHD [50], Keuppens and colleagues developed a CBT-based sleep intervention specifically for adolescents with ADHD [52]. The intervention – Sleep IntervEntion as Symptom Treatment for ADHD (SIESTA) – incorporates sleep hygiene practices, cognitive-behavioral principles (e.g., stimulus control), and planning and organizational skills using motivational interviewing, and is comprised of six sessions with the adolescent and two sessions with caregivers. Initial qualitative and quantitative pilot work provided preliminary support for intervention satisfaction and improvements in sleep hygiene [52]. A subsequent RCT involving 92 (ages 13–17 years) adolescents with ADHD and sleep problems (defined as short sleep duration, long sleep onset latency, or long night awakenings, in addition to one inadequate sleep hygiene practice) compared SIESTA with treatment as usual (TAU) to TAU alone, with ratings and actigraphy collected at pre-intervention, post-intervention, and at a four-month follow-up [51]. Compared to TAU, participants randomized to SIESTA + TAU showed significant pre-post improvement in sleep hygiene, chronic sleep reductio, and sleep-wake problems. There was also a pre-post reduction in the secondary outcome of self-reported depressive symptoms favoring SIESTA + TAU. However, there were no intervention effects for actigraphy or daily diary outcomes, nor for self-reported daytime sleepiness, caregiver-reported sleep difficulties, or other secondary outcomes (ADHD and other mental health symptoms; caregiver-adolescent conflict; homework and classroom performance). Given these mixed findings alongside minimal effects at the four-month follow-up, there is a need for additional research to examine subgroups of adolescents with ADHD who may be more likely to benefit from SIESTA, studies comparing ADHD-adapted behavioral sleep interventions to existing CBT-based sleep interventions, incorporating additional CBT-insomnia strategies (e.g., sleep restriction) and circadian-based approaches, and evaluating the value-add of booster sessions or other strategies (e.g., text messaging) to support maintenance [51].

Considered together, across early and middle childhood and adolescence, there is growing and promising evidence to support behavioral sleep interventions in youth with or at risk for ADHD. These interventions typically result in improvements in parent-rated sleep problems. Although this finding is robust, its value is limited somewhat by use of passive rather than active control groups and by the fact that blinding cannot be applied in these studies due to the parent-focused nature of the treatments. Less consistently, this literature finds improvements with intervention in objective sleep metrics, ADHD symptoms, psychosocial functioning, and quality of life. Additional research is needed to further test and refine interventions, evaluate theoretically grounded mechanisms that may be particularly potent intervention targets, consider individual and contextual factors that may moderate effects, and leverage approaches that can support sustained improvements to improve sleep and associated impairments over time.

### Integrating and Evaluating Sleep in Evidence-Based Behavioral Interventions for ADHD

In addition to the importance of work focused specifically on behavioral sleep interventions for youth with ADHD, another approach is to examine the extent to which existing evidence-based behavioral interventions developed and tested to improve the daytime functioning of youth with ADHD also improve sleep functioning, or whether sleep difficulties predict or moderate treatment response to ADHD-based behavioral interventions. Despite numerous evidence-based reviews and clinical guidelines for ADHD, sleep is seldom addressed as a co-occurring condition. For example, the 2019 American Academy of Pediatrics [53] clinical practice guideline mentions screening for sleep apnea and notes sleep disturbance as a possible side effect of stimulants but does not cover behavioral sleep issues. Similarly, the 2020 Society of Developmental and Behavioral Pediatrics [54] clinical practice guideline focused on complex ADHD (including co-occurring conditions) briefly acknowledges sleep disorders but excludes them due to space limitations. Although these guidelines are highly valuable, they illustrate that sleep disturbances remain underrepresented in ADHD assessment and treatment despite their high prevalence, in part due to limited empirical evidence to support strong recommendations. Accordingly, in this section we review the limited research examining the impact of evidence-based behavioral interventions for ADHD on sleep outcomes or the incorporation of sleep-based strategies into ADHD-based interventions, again using a developmental approach given evidence-based behavioral interventions for both ADHD [53, 54] and sleep [55, 56] differ across early childhood, middle childhood, and adolescence.

#### Early Childhood

The primary behavioral treatment for preschoolers with ADHD is behavioral parent training (BPT). BPT is implemented with the caregivers of young children and utilizes the antecedent-behavior-consequence (ABC) framework and operant conditioning to help caregivers shape a child’s behavior [53]. Antecedent strategies include provision of clear expectations and rules, effective instructions, and implementation of consistent routines and schedules, while consequences emphasize increasing positive behaviors though rewards and reducing negative behaviors through the removal of reinforcing stimuli (e.g., parental attention) or effective punishments [57]. Notably, behavioral sleep interventions also rely on the ABC framework [58], with a primary difference between BPT and behavioral sleep interventions centered on whether improving daytime versus nighttime/sleep behaviors are the primary target of intervention [24].

Sleep disturbances are rarely a reported outcome of BPT trials in preschoolers with elevated ADHD symptoms, even when sleep is briefly addressed as part of the BPT intervention (e.g [59]), . However, clinical trials of Parent-Child Interaction Therapy (PCIT), a specific version of BPT that promotes enhanced caregiver–child interactions and responsive, consistent parenting practices, suggests BPT may result in improved sleep in related preschool populations. For example, among 44 children aged 20–70 months with developmental delay and co-occurring externalizing behavior problems, PCIT was found to decrease parent-reported sleep problems compared to a waitlist control [60]. More recently, a PCIT trial among 403 children ages 2–9 with disruptive behaviors found pre-post intervention improvements in parent-reported bedtime resistance, sleep onset delay, sleep-related anxiety, sleep duration, nocturnal awakenings, and parasomnias [61]. Importantly, while BPT interventions may improve sleep in preschoolers, the mechanisms by which it does so remain unclear [61].

#### Middle Childhood

A small number of studies of behavioral treatments for ADHD have evaluated sleep changes in response to these treatments, despite the intervention not being focused on sleep specifically. Findings of these studies are mixed. A post hoc analysis of the Multimodal Treatment for ADHD (MTA) study data indicated that the intensive behavioral treatment provided as part of that study did not significantly impact sleep outcomes [62]. The behavioral treatment provided in the MTA trial encompassed behavioral parent training (27 group sessions + 8 individual sessions), child-focused treatment (an 8-week daily summer program reinforcing behavior management and providing social skills and study skills training + 3 months of individual behavior management training), and school-based intervention that consisted of 3–4 months of regular teacher consultation around implementing classroom behavior management strategies. Despite the intensive nature of this treatment program, reduction in parent-rated sleep problems did not reach significance. Similarly, an online psychosocial program for children with ADHD and autism, consisting of 12 weekly 90-minute virtual sessions covering psychoeducation and stress management, cognitive-behavioral techniques, and social and communication skills, did not improve sleep problems among participants despite improving stress, coping, and social support [63].

A unique intervention, based on BPT, but adapted to address screen time, exercise, and sleep for youth with ADHD, has been tested against BMT alone and offers some insights on sleep. The Lifestyle Enhancement for ADHD Program (LEAP) [64] is a 9-week remotely-administered group intervention for parents of youth with ADHD. Weekly 90-minute sessions covered psychoeducation around ADHD and health behaviors (including sleep hygiene), mindfulness practice, and instruction in behavior management principles and techniques. In a RCT comparing LEAP to traditional BMT, the two interventions had similar effects on sleep: neither treatment changed sleep duration significantly, and both treatments resulted in less parent-reported bedtime resistance immediately following treatment, but gains were not sustained 10 weeks later in either group. Notably, ADHD symptoms and executive functions did improve post-intervention for both groups, despite sleep being unaffected [64].

#### Adolescence

Only two studies have examined sleep in the context of behavioral interventions for adolescents with ADHD.

In a RCT examining the school-based Challenging Horizons Program (CHP; *n* = 85) compared to a community care condition (*n* = 86) in high school students (grades 9–11), sleep hygiene and daytime sleepiness were examined as predictors of academic and organization intervention response [65]. Prior to treatment, adolescents completed measures of sleep hygiene and daytime sleepiness. In addition, a component of the high school version of the CHP intervention included 10 coach-led after-school parent sessions focused primarily on the development of a home-work management plan but also included some focus on sleep. Specifically, Session 2 focused on sleep hygiene practices, including both daytime (e.g., regular exercise, reduced caffeine use) and nighttime activities (e.g., bedtime routine, consistent sleep schedule) that promote healthy sleep. Implementation of strategies for improving sleep hygiene were discussed and reviewed as a group in Session 3, and Session 6 included a review of well-being in adolescence and its connection to sleep hygiene.

In separate models examining sleep hygiene and daytime sleepiness as predictors of post-treatment outcomes, both poorer sleep hygiene and higher daytime sleepiness were associated with lower post-treatment grade point average (GPA) obtained from school records, poorer parent-reported organizational skills, and more parent-reported academic and homework problems (pre-intervention academic/organization scores were not included). However, only one interaction with treatment group was significant: higher pre-treatment daytime sleepiness was associated with more homework problems only for adolescents in the community care condition. The authors interpreted this as indicating a buffering effect wherein adolescents in the CHP group were protected from the negative impact of daytime sleepiness for homework functioning [65].

As attendance at the parent sessions was variable (median: 4, range: 0–10), it is unknown which parents received the information on sleep hygiene and, among those who received this information, whether they discussed or implemented any of the sleep hygiene practices with their adolescent. In addition, the sleep hygiene information was provided in parent groups but was not incorporated into the individual or group sessions with adolescents themselves, which may be important to engage adolescents with ADHD in sleep-related treatment goals and to address barriers and facilitators to implementing healthy sleep practices. These are important directions for future research examining the extent to which sleep and/or sleepiness domains predict or moderate response to academic and organization-focused interventions for adolescents with ADHD.

The second study that examined sleep in the context of an intervention directed to adolescents with ADHD was a small (*n* = 14) open trial of the Mindful Awareness Practices (MAPs) program [66]. MAPs consists of eight sessions delivered in a group format, and although sleep is not a direct focus, the emphasis on mindfulness practices that may support emotion regulation and interrupt maladaptive thinking patterns may have downstream improvements for sleep. However, no pre-post change was observed in adolescent-report of sleep hygiene. As with the CHP study [65], it will be important for future research to include a broader measurement of sleep, including sleep domains (e.g., sleep onset, quality, duration) and assessment methods (e.g., diary, actigraphy). Of note, when asked for suggestions that may improve the MAPs program, numerous adolescents and parents suggested more information on sleep, including incorporating mindful practice in bedtime routine, and education on healthy sleep habits. Directly integrating sleep into mindfulness-based interventions may prove beneficial to adolescents with ADHD across numerous domains of functioning.

In summary, on the basis of the very limited literature, we do not have evidence to suggest that empirically-supported treatments for ADHD in children and adolescents, such as parent BMT, are effective, in and of themselves, in improving sleep health for these children. Although components of BMT are often infused in sleep-focused treatments for children and adolescents with ADHD, other common ingredients, such as bedtime fading, stimulus control, and strategies targeting adolescent-delivered sleep hygiene practices, may be critical to the success of these treatments.

### Emerging Directions

Building on the growing evidence base for behavioral sleep interventions in youth with ADHD, the field is now poised to move beyond demonstrating efficacy toward refining, personalizing, and scaling these approaches. Emerging directions reflect a shift toward more intentional integration of sleep within ADHD treatment frameworks, increased attention to developmental and mechanistic processes, and the use of innovative delivery models to enhance reach and sustainability. At the same time, recent reviews highlight the need for more precise characterization of sleep disturbances, rigorous and pragmatic randomized trials, and greater focus on long-term and functional outcomes beyond sleep [2, 19]. In this section, we highlight key areas of advancement across developmental periods, as well as cross-cutting approaches that have the potential to transform how sleep is assessed and treated within ADHD care.

#### Early Childhood

Given the dearth of research on behavioral sleep interventions in preschool ADHD populations to date, it is exciting to note two clinical trials currently underway that are designed to address this key gap in the literature. Preschool Sleep and Attention Support (PASS) is an 8-week intervention that streamlines BPT (4 sessions) with behavioral sleep intervention (4 sessions) for preschoolers aged 3–5 with elevated clinician-rated ADHD symptoms and parent-reported sleep disturbances [67]. The two intervention elements are implemented sequentially, with BPT provided first to orient the caregiver to the ABC framework/operant conditioning principles and target daytime behaviors, followed by the behavioral sleep intervention, which teaches caregivers to apply the ABC framework to shape bedtime and sleep-related behaviors as well as specific sleep strategies (e.g., consistent sleep/wake schedule and bedtime routines, bedtime fading, skills to support independent sleeping). PASS is delivered in an individual-family, telehealth-delivered therapy format by social workers working in behavioral health clinic settings. The current trial underway will compare PASS to a standard 8-session BPT for improving preschoolers’ sleep, ADHD severity, comorbid symptoms, and functional impairment.

The Optimizing Attention and Sleep Intervention Study (OASIS) is a 6-session, individual-family format, telehealth-delivered intervention that also streamlines BPT and behavioral sleep intervention principles into a single intervention for preschoolers aged 3–5 with attentional and sleep concerns [68], but unlike PASS, each session of OASIS includes components of both BPT and behavioral sleep intervention. Another notable difference between OASIS and PASS is that social workers delivering OASIS are working in integrated pediatric primary care, rather than behavioral health settings. The OASIS trial will compare its streamlined intervention to an abbreviated, developmentally-appropriate 6-session BPT-only intervention and will investigate relative improvements in sleep (including nighttime awakenings, sleep onset latency, and consistent in bedtime routine) as well as ADHD severity across the conditions. Findings from both PASS and OASIS trials will provide critical new information regarding the impact of behavioral sleep interventions for improving ADHD symptoms in early development, and whether such interventions outperform the current gold-standard intervention for this age group (i.e., BPT).

#### Middle Childhood

Because ADHD is a multifaceted and heterogeneous condition, it stands to reason that treatments for ADHD should offer varied and individualized approaches. With the increasing acknowledgement of ADHD’s impact on nighttime as well as daytime functioning, evidence-based strategies to treat sleep problems will need to be incorporated into treatment protocols for these children. At this point, we have several empirically-supported behavioral options for treating daytime ADHD symptoms and associated impairments, including BPT to address problem behaviors, social skills groups to address the social challenges that many children with ADHD face, and organizational skills training to address executive dysfunction [53, 54]. For a child who demonstrates problem behaviors and social difficulties, we might use both behavior management training and social skills training in their treatment. Similarly, we now have evidence to support the effectiveness of strategies such as bedtime fading, stimulus control, and psychoeducation in sleep hygiene in reducing nighttime problems associated with ADHD.

Although untested to date, an individualized, modular approach to behavioral ADHD treatment, may have benefits for both effectiveness and engagement. Such an approach could begin with a thorough assessment of the child’s symptoms and functioning to inform which ‘modules’ of treatment would be most useful for them and to prioritize treating the issues that are currently causing the greatest functional impairment first. If daytime problem behaviors are prioritized, thorough training of parents in behavior management would come first, whereas if sleep onset difficulties are primary, a brief overview of foundational behavior management techniques might be quickly followed by specific instruction in bedtime fading or stimulus control. Using a collaborative shared decision-making process [69, 70], clinicians could have a menu of evidence-based behavioral treatment components addressing both daytime and nighttime functioning and, following some foundational and cross-cutting skills training, they would be able to work with caregivers (and adolescents themselves) to select and prioritize the components that are most relevant for their specific child.

The success of this type of modular approach rests on a strong evidence base for behavioral strategies to treat the wide variety of challenges that children with ADHD may face during the day and at night. The cornerstones have been laid for this foundation, but there remain several behavioral approaches to sleep treatment for children with ADHD that are promising in theory but require additional testing. Meditation and mindfulness strategies are increasingly being tested with youth with ADHD and may be particularly well-suited to the treatment of sleep problems. The Power Down intervention [71], for example, integrates gentle pressure massage with a mindfulness protocol and is currently being tested in children with ADHD and difficulty falling asleep. This intervention is accomplished with a 1-hour in-person session followed by two weeks of implementation of the bedtime protocol each night. The bedtime protocol consists of the parent providing a gentle pressure massage paired with verbal cues to “power down” each successive muscle during the massage in order to relax and ready themselves for sleep. Lifestyle approaches addressing physical activity and screen use as means to improve sleep in school-aged children with ADHD also seem promising and warrant further investigation [72]. As the field continues to identify behavioral strategies that work to treat ADHD, these can be added to the toolbox in the hopes of being able to offer treatments that address the full spectrum of the daytime and nighttime symptoms that children with ADHD experience.

#### Adolescence

During adolescence, advancing the treatment of sleep in ADHD will require more explicit integration of circadian science, sleep regularity, and developmentally appropriate delivery approaches that align with adolescents’ increasing autonomy. Adolescents show a normative shift toward later circadian phase preference, heightened eveningness, and increased variability in sleep timing [47], all of which may be more pronounced in adolescents with ADHD [17, 73-75]. Accordingly, interventions that extend beyond traditional sleep hygiene and insomnia-focused cognitive-behavioral strategies to directly target circadian timing and sleep regularity represent a particularly promising direction. One such approach is the emerging focus on promoting consistent sleep-wake patterns. Sleep regularity interventions, such as the ongoing Sleep Health Enhancement to Improve Teens’ Sleep (SHEETS; NIMH Grant R34MH128440) protocol, target day-to-day variability in sleep timing as a mechanism for improving both sleep and daytime functioning. SHEETS, a 6-week digital (smartphone app) intervention, seeks to improve sleep regularity through education activities, behavioral change, and biometric feedback (real-time communication about sleep regularity, measured by a Garmin watch, communicated to the adolescent through the app). Although SHEETS was not designed specifically for adolescents with ADHD, anticipated outcomes of the current clinical trial will include the effect of SHEETS on improving psychiatric health, including ADHD symptoms. This approach may be particularly well-suited for adolescents with ADHD who are prone to experiencing irregular schedules, behavioral challenges, and other difficulties (e.g., homework problems) that may interfere with maintaining a regular sleep-wake schedule.

Interventions that directly target circadian phase delay are also needed. Bright light therapy and related chronobiological approaches have demonstrated efficacy in shifting circadian timing and improving sleep in adolescents and may have downstream benefits for ADHD symptoms, though they remain understudied in ADHD-specific samples [49]. Combining circadian-based approaches with CBT-based sleep interventions may yield synergistic effects, particularly for adolescents with pronounced eveningness or delayed sleep phase patterns [76, 77].

Another key direction is leveraging developmentally appropriate engagement strategies. Adolescents with ADHD often exhibit reduced motivation, reward sensitivity differences, and difficulties with planning and follow-through that may limit engagement and adherence to behavioral sleep recommendations. Drawing from ADHD-developed interventions such as Supporting Teens’ Autonomy Daily (STAND) [78], it would beneficial for sleep-focused approaches to incorporate motivational interviewing, reminders, and contingency management strategies that support engagement and may sustain behavior change over time.

#### Spanning Development and Increasing Access

Across developmental periods, a central challenge and opportunity is to increase access to effective behavioral sleep interventions for youth with ADHD while tailoring approaches to developmental needs and contexts. One promising avenue is the integration of sleep assessment and intervention within existing service delivery systems, including integrated behavioral health (IBH) models in pediatric primary care. Given that many youth with ADHD are first identified and treated in primary care settings, embedding brief, scalable sleep interventions into these settings could substantially increase reach and reduce barriers to care. Emerging trials such as OASIS illustrate the feasibility of delivering streamlined behavioral sleep and ADHD interventions in primary care [68].

Schools also represent a critical context for identifying and addressing sleep difficulties, particularly in middle childhood and adolescence. School-based interventions for ADHD that target academic and organizational functioning may benefit from incorporating sleep education and intervention components, especially given evidence that sleep and daytime sleepiness predict academic outcomes and treatment response [65]. Although research in this area remains limited, integrating sleep-focused modules into school-based programs such as the Challenging Horizons Program (CHP) [65] or Homework, Organization and Planning Skills (HOPS) [79] may enhance their effectiveness and ecological validity.

Technological innovations offer additional opportunities to expand access and personalize care (e.g [80]), . Telehealth and fully digital interventions, such as Better Nights, Better Days, demonstrate that sleep interventions can be delivered effectively with minimal clinician involvement [40]. These approaches may be particularly valuable for families facing geographic, financial, or logistical barriers to care. Although not yet tested in ADHD samples, low-burden text messaging interventions may be beneficial for promoting maintenance of behavioral sleep treatment effects [81]. Advances in ambulatory sleep monitoring, including wearable devices and actigraphy, may further enhance intervention delivery by providing objective, real-time data on sleep patterns. Although challenges remain regarding accuracy and interpretation, especially in pediatric populations, these tools may facilitate personalized feedback, self-monitoring, and adaptive intervention strategies [82, 83]. As one promising example, it has been proposed that wearable sensors may be a feasible approach to elucidate circadian dysfunction in ADHD heterogeneity, supporting personalized sleep-circadian treatments [84]. Integrating wearable data with intervention platforms could support precision medicine approaches, tailoring treatment components based on individual sleep phenotypes and response patterns. Forthcoming results from current clinical telehealth and/or digital intervention trials, such as PASS, OASIS, and SHEETS, will further inform these possibilities. It will be especially important to learn from these trials if incorporating digital health technologies improves outcomes across subjective and objective sleep methods, particularly given the limited evidence reviewed above of behavioral sleep interventions to improve objectively-measured sleep indices among youth with ADHD.

Combined and sequential treatment approaches also warrant further investigation. For example, pairing behavioral sleep interventions with pharmacological treatments such as melatonin may enhance outcomes for some youth, particularly those with circadian phase delays or persistent sleep onset difficulties [2]. Similarly, determining the optimal sequencing (i.e., whether it is best to treat sleep or ADHD first, or target both simultaneously), dosage, and intensity of interventions across development remains an important direction for future research. Finally, incorporating the perspectives of youth with ADHD and their families through lived experience frameworks and participatory action approaches will be essential to ensure that sleep interventions are acceptable, feasible, and responsive to real-world needs.

## Conclusions

Sleep disturbances are highly prevalent, persistent, and impairing among youth with ADHD, and they represent a critical yet historically under-addressed treatment target. Over the past decade, the field has made meaningful progress in developing and testing behavioral sleep interventions for children and adolescents with ADHD, with particularly encouraging findings for improving parent-reported sleep problems and some aspects of psychosocial functioning [19, 20]. At the same time, important gaps remain, including limited effects on objective sleep indices, variable generalization to daytime functioning, and challenges related to sustaining treatment gains over time [19, 20].

A developmental perspective is essential for advancing research and clinical care in the area of behavioral sleep intervention and ADHD. Looking forward, progress will depend on several key priorities. First, there is a need for larger, well-powered randomized controlled trials that incorporate rigorous measurement of both subjective and objective sleep outcomes, as well as downstream effects on ADHD symptoms and functioning [2]. Second, identifying mechanisms of change and treatment moderators will be critical for optimizing and personalizing interventions. Third, efforts to integrate sleep into existing ADHD care pathways, including primary care, schools, and digital platforms, will likely be essential for increasing access and reach. Finally, embracing innovative approaches alongside participatory engagement, including circadian-based interventions, wearable technologies, and modular or precision medicine frameworks, holds promise for delivering more effective and individualized care. Together, advances in each of these areas have the potential to meaningfully improve both nighttime and daytime functioning for children and adolescents with ADHD and their families.

## Data Availability

No datasets were generated or analysed during the current study.
